# Diterpenoids from Roots of *Salvia lachnocalyx;* *In-silico* and *In-vitro* Toxicity against Human Cancer Cell Lines

**DOI:** 10.22037/ijpr.2019.15429.13095

**Published:** 2020

**Authors:** Hossein Hadavand Mirzaei, Omidreza Firuzi, Amir Reza Jassbi

**Affiliations:** a *Medicinal and Natural Products Chemistry Research Center, Shiraz University of Medical Sciences, Shiraz, Iran. *; b *Department of Molecular Physiology, Agricultural Biotechnology Research Institute of Iran, Agricultural Research, Education and Extension Organization (AREEO), Karaj, Iran.*

**Keywords:** Salvia lachnocalyx, Diterpenoids, Cytotoxic activity, Molecular docking

## Abstract

Further investigations on phytochemical constituents of dichloromethane extract from roots of *Salvia lachnocalyx* (*S. lachnocalyx)* led to the isolation and identification of eight known diterpenoids from this plant for the first time. The chemical structures of the purified compounds were elucidated using spectroscopic analyses including EI-MS, ^1^H and ^13^C NMR and by comparison of the resulting spectra with those reported in the literature. Then, the cytotoxic activity of identified compounds was examined against two human cancer cell lines MCF-7 (human breast adenocarcinoma) and K562 (human chronic myelogenous leukemia). Molecular docking of promising cytotoxic compounds were performed by AutoDock Tools 1.5.4 program in the active site of Topoisomerase I. Eight known diterpenoids; 12-hydroxysapriparaquinone (**1**), 15-deoxyfuerstione (**2**), horminon (**3**), 7α-acetoxyroyleanone (**4**), 11β-hydroxymanoyl oxide (**5**), microstegiol (**6**), 1-keto-aethiopinone (**7**) and 14-deoxycoleon U (**8**) were isolated of dichloromethane extract from roots of *salvia lachnocalyx*. Compounds **2**, **3**, **6,** and **8** showed cytotoxic activity against MCF-7 (human breast adenocarcinoma) and K562 (human chronic myelogenous leukemia) cell lines with IC_50 _values in the range of 2.63-11.83 µg/mL. The inhibition of” topoisomerase I” was suggested by molecular docking calculations as the mechanism of cytotoxicity of the tested compounds. According to cytotoxic assay and docking results, it is suggested that compounds **2**, **3**, **6**, and **8** have good potential as anticancer agents.

## Introduction

*Salvia *(sage) is an important genus among the medicinal plants of the family *Lamiaceae* with about 900 species spread throughout the world. A literature survey demonstrated that *Salvia* species have been reported for treatment of many of different diseases. Abitane, rearranged abitane and tanshinones are principal secondary metabolites of the roots of *Salvia* species and reported with diverse pharmacological activities (1, 2).

On continuing of our research on separation and structure elucidation of the bioactive compounds from Iranian *Salvia* species, the DCM extract of shoots and roots of *S. lachnocalyx* have been selected for further investigation based on primary results of cytotoxic screening of different *Lamiaceae and Solanaceae *plants (3). 

*S. lachnocalyx* with the Persian name “Maryam-goli Eghlidi” is an endemic plant of Iran growing in the Fars province (4). Previous phytochemical studies of *S. lachnocalyx* have revealed the presence of five sesterterpene lactones (lachnocalyxolide A-C, salvileucolide methyl ester and salvileucolide-6,23-lactone), three flavonoids (salvigenin, eupatorin and cirsilio), two steroidal compounds (*β*-sitosterol and daucosterol), one nor diterpenoid (norambreinolide-18, 6*α*-olide) and one triterpenoid (ursolic acid) in the acetone extract of the aerial parts. In addition to the above phytochemical analyses, the cytotoxic potential of isolated sesterterpene lactones have been assessed against two human cancer cell lines (MCF-7 and HeLa) and no cytotoxic activity has been reported for the tested compounds (IC_50_ > 50 μM) (5). In another study, the GC-MS profile of the essential oil of *S. lachnocalyx*, collected from Fras province, has been recorded and the main components of the oil were reported as bicyclogermacrene (31.3%), α-pinene (13.2%), sabinene (11.7%), and β-pinene (10.3%) (6).

We have subjected the shoots extract of *S. lachnocalyx* to a cytotoxic bioassay-guided fractionation experiments and separated two cytotoxic compounds; (2Z,6Z,10Z,14E)-geranylfarnesol and spathulenol with IC_50_ values ranging from 9.6 to 20.2 µg/mL against three human cancer cell lines (3). Recently, we have separated five diterpnoids including ferruginol, taxodione, sahandinone, 4-dehydrosalvilimbinol and labda-7,14-dien-13-ol with labdane, abietane, and rearranged-abietane diterpenoid skeletons from a DCM extract of *S. lachnocalyx*. The isolated compounds showed significant cytotoxicity against three human cancer cell lines (IC_50_ range: 0.41-17.23 µg/mL) (7). In the present study we report more cytotoxic compounds from the polar fractions obtained from silica gel column chromatography of the DCM roots extract of *S. lachnocalyx*. 

Assessment of the mode of action of natural and synthetic compounds by using computational approach is being increasingly exploited, because of this method being faster and cheaper than lab experiments (8). Docking studies is applied in both the rational drug design and mechanistic assessment by evaluating mode of interaction, binding affinity and orientation of a ligand into active site of the macromolecular target. Therefore, identification of therapeutic target has a key role in the discovery of new drugs (9).

In this study, in addition to isolation of further compounds from the roots extract of *S. lachnocalyx,* their cytotoxicity and possible mechanism of action have been examined by docking experiments.

## Experimental

*Reagents and chemicals*

Fetal bovine serum (FBS), phosphate buffered saline (PBS), RPMI 1640, and trypsin were purchased from Biosera (Ringmer, UK). Acetonitrile (ACN, HPLC grade), dichloromethane (DCM), dimethyl sulfoxide (DMSO), methanol (MeOH), silica gel (70–230 mesh) for open column chromatography and pre-coated silica gel F_254_ TLC aluminum sheets were obtained from Merck (Darmstadt, Germany). 3-(4,5-Dimethylthiazol-2-yl)-2,5-diphenyltetrazolium bromide (MTT) was provided from Sigma-Aldrich (St. Louis, MO, USA), while cisplatin and penicillin/streptomycin were provided from EBEWE Pharma (Unterach, Austria).

*Instrumentation*

NMR spectra were recorded on a Bruker Avance 300 MHz spectrometer operating at 300.13 MHz for ^1^H and 75.4 MHz for ^13^C. Mass spectra (EI-MS) were recorded on an Agilent 5975C inert GC/MSD instrument. 

Purification of the fractions obtained from silica gel open column were performed using preparative RP-HPLC on a Knauer system consisting of a K-1800 pump, with an RP C18 (Eurospher II 100-5 C18, 250 × 20 mm ID with pre-column 30 × 20 mm ID) column. The mobile phase consisted of acetonitrile water (ACN/H_2_O: 90:10) for purification of Fr12 and ACN/H2O (80:20) for Fr17 and Fr18. The K-2500 UV–Vis detector was set at 210 nm. Each of the pure compounds was manually collected from outlet of the prep-HPLC column guiding the recording chromatogram. 

*Plant material*

The roots of *Salvia lachnocalyx *Hedge were collected from Eghlid in the Fars Province, Iran, in May 2015 and identified by Mojtaba Asadollahi, plant taxonomist at the Medicinal and Natural Product Chemistry Research Center (MNCRC). A voucher specimen (No. 94-3-8-4) was preserved at the herbarium of MNCRC, Shiraz University of Medical Sciences, Fars, Iran.

*Extraction and isolation*

The extraction and fractionation procedure of the roots extracts was described previously (7). Briefly, the dried root samples were ground and the resulting ground plant material (400 g) were extracted with DCM successively (3 × 2 L) at room temperature for 48 h. The extracts were evaporated under reduced pressure at a temperature 40 °C to yield 6.0 g of a dark red residue. The concentrated DCM extract was fractionated over a silica gel (110 g, 70-230 mesh) column chromatography (100 × 5 cm) The column was eluted with a gradient of *n*-hexane →CH_2_Cl_2_ (100/0 to 0/100%) followed by CH_2_Cl_2_→MeOH (100/0 to 0/100%). Forty-nine fractions (300 mL each) were collected and pooled into 22 fractions (Fr 1–22) according to the similarity of their TLC chromatogram pattern (mobile phase CH_2_Cl_2_/EtOAc 8:2). Analytical and preparative RP C18 HPLC analyses of Fr12, 17 and 18 resulted in purification of compounds **1-8**. Compounds **1** (4.0 mg, R_t_ = 21.5 min) and **2** (6.6 mg, R_t_ = 24.45 min) were isolated from Fr 12. Compounds **3 **(21.0 mg, R_t_ = 9.8 min), **4** (13.6 mg, R_t_ = 11.5 min), **5 **(7.5 mg, R_t_ = 20.9 min) and **6 **(9.1 mg, R_t_ = 24.9 min) were purified from Fr 17 and Finally compounds **7** (3.3 mg, R_t_ = 10.1 min) and **8** (17.7 mg, R_t_ = 12.3 min) were isolated from Fr 18. 

*Cell lines and culture*

MCF-7 (human breast adenocarcinoma) and K562 (human chronic myelogenous leukemia) cell lines were provided from Iranian Biological Resource Center, Tehran, Iran. The cells in monolayer were seeded in sterile T25 flasks in RPMI 1640 medium supplemented with 10% v/v fetal bovine serum, penicillin (100 units/mL) and streptomycin (100 µg/mL) and the flasks were incubated at 37 °C in a 5% CO_2_ incubator. 

*Cytotoxicity assay*

The cytotoxic activity was examined using the MTT reduction assay (10). In this colorimetric assay, the yellow color of tetrazolium bromide (MTT) is converted to the purple color of formazan by the action of mitochondrial dehydrogenase enzymes in viable cells. The dried compounds with suitable purity (≥ 95%) were dissolved in DMSO to obtain stock solution and then diluted in growth medium at least 400 times. The cells were added to each well of 96-well plates at the density of 50,000 cells/mL in 100 µL of growth medium and the plates were incubated at 37 °C for 24 h. After incubation, 50 µL of the medium was replaced with 50 µL of test compounds diluted in fresh growth medium (3-4 different concentrations) and incubation continued for a further 72 h. Then, the medium of each well was removed and replaced by RPMI without phenol red containing MTT 0.5 mg/mL and incubated for an additional 4 h. DMSO was used to solubilize the formed formazan crystals. The absorbance of the wells was measured at 570 nm, with background correction at 655 nm using a microplate reader and percentages of antiproliferative activity was calculated compared to the untreated control wells. IC_50_ was calculated from the sigmoidal growth inhibition curves using CurveExpert software, version 1.3 for Windows.

*Spectroscopic data of the purified compounds*

12-Hydroxysapriparaquinone (**1**): C_20_H_24_O_3_; ^1^H NMR (CDCl_3_, 300.13 MHz): δ = 7.96 (1H, d, *J* = 7.8 Hz, H-7), 7.51 (1H, d, *J* = 7.8 Hz, H-6), 5.29 (1H, t, *J* = 9.0 Hz, H-3), 3.36 (1H, heptet, *J* = 7.1 Hz, H-15), 3.19 (2H, m, H-1), 2.45 (3H, s, H-20), 2.20 (2H, m, H-2), 1.73 (3H, s, H-18), 1.61 (3H, s, H-19), 1.30 (6H, d,* J* = 7.1 Hz, H-16,17); El-MS: *m/z *(rel int.): 312[M]^ +^ (100), 295 (7), 244 (25), 229 (5), 201 (5), 129 (4), 75 (100), 69 (16), 41 (10). 

15-deoxyfuerstione (**2**): C_20_H_26_O_2_; ^1^H NMR (CDCl_3_, 300.13 MHz): δ = 7.73 (1H, *br* s, OH-11), 6.92 (1H, s, H-14), 6.74 (1H, d, *J* = 6.7 Hz, H-7), 6.37 (1H, d, *J* = 6.7 Hz, H-6), 3.30 (1H, m, H-1a), 3.17 (1H, heptet, *J* = 6.9 Hz, H-15), 1.98 (1H, ddd, *J* = 17.5, 11.3, 4.4 Hz, H-2a), 1.66 (1H, m, H-3a), 1.62 (1H, m, H-2b), 1.56 (3H, s, H-20), 1.52 (1H, ddd, *J* = 13.1, 7.5, 4.2, Hz, H-1b), 1.42 (1H, dt,* J* = 11.8, 4.5 Hz, H-3b), 1.30 (3H, s, H-19), 1.22 (3H, s, H-18), 1.19 (3H, d, *J *= 7.0 Hz, H-16), 1.18 (3H, d, *J* = 7.0 Hz, H-17); ^13^C NMR (CDCl_3_, 75.4 MHz): δ = 178.2 (C-12), 167.9 (C-11), 146.2 (C-5), 141.3 (C-13), 138.9 (C-7), 133.0 (C-14), 127.3 (C-8), 128.3 (C-9), 118.1 (C-6), 42.9 (C-10), 40.9 (C-3), 37.9 (C-4), 34.3 (C-1), 32.9 (C-18), 30.0 (C-19), 26.9 (C-15), 24.7 (C-20), 21.9 (C-16), 21.7 (C-17), 18.6 (C-2); El-MS: *m/z *(rel int.): 298 [M]^ +^ (30), 283 (5), 255 (2), 242 (15), 229 (100), 201 (9), 115 (4), 55 (4), 41 (5).

Horminone (**3**): C_20_H_28_O_4_; ^1^H NMR (CDCl_3_, 300.13 MHz): δ = 7.27 (1H, s, OH-12), 4.72 (1H, dd, *J* = 4.0, 1.5 Hz, H-7), 3.14 (1H, heptet, *J* = 6.9 Hz, H-15), 3.05 (1H, *br* s, OH-7), 2.68 (1H, ddd,* J* = 14.0, 4.0, 4.0 Hz, H-1β), 1.94 (1H, *br* d, *J* = 13.5 Hz, H-6α), 1.73 (1H, m, H-2β), 1.60 (1H, ddd,* J* = 13.5, 13.2, 4.4 Hz, H-6β), 1.56 (1H, m, H-2α), 1.48 (1H, m, H-5),1.34 (1H, m, H-3β), 1.24 (1H, m, H-3α), 1.20 (3H, s, H-20), 1.21 (3H, d, *J *= 6.9 Hz, H-17), 1.19 (3H, d, *J* = 6.9 Hz, H-16), 1.13 (1H, *br* d,* J* = 4.0 Hz, H-1α), 0.97 (3H, s, H-19), 0.89 (3H, s, H-18).^13^C NMR (CDCl3, 75.4 MHz): δ =189.1 (C-14), 183.9 (C-11), 151.1 (C-12), 147.8 (C-9), 143.1 (C-8), 124.2 (C-13), 63.2 (C-7), 45.7 (C-5), 41.08 (C-3), 39.1 (C-10), 35.7 (C-1), 33.2 (C-18), 33.0 (C-4), 25.7 (C-6), 23.9 (C-15), 21.7 (C-19), 19.8 (C-16), 19.7 (C-17), 18.8 (C-2), 18.3 (C-20); El-MS: *m/z *(rel int.): 332 [M]^ +^ (100), 314 (94), 298 (85), 283 (40), 267 (66), 227 (65), 183 (27), 128 (18), 55 (18), 43 (32). 

7 α -acetoxyroyleanone (**4**): C_22_H_30_O_5_; ^1^H NMR (CDCl_3_, 300.13 MHz): δ = 7.15 (1H, s, OH-12), 5.95 (1H, dd, *J* = 3.7, 1.4 Hz, H-7), 3.18 (1H, heptet, *J* = 7 Hz, H-15), 2.73 (1H, *br* d, 13.1 H-1β), 2.03 (3H, s, H-22), 1.94 (1H, *br*d, *J* = 14.5 Hz, H-6α), 1.78 (1H, m, H-2β), 1.70 (1H, m, H-6β), 1.59 (1H, m, H-2α), 1.52 (1H, m, H-3β), 1.46 (1H, t, *J* = 3.6 Hz, H-5), 1.29 (1H, m, H-3α), 1.23 (3H, s, H-20), 1.22 (1H, m, H-1α), 1.21 (3H, d, *J *= 7.0 Hz, H-17), 1.18 (3H, d, *J* = 7.0 Hz, H-16), 0.88 (3H, s, H-18), 0.87 (3H, s, H-19). ^13^C NMR (CDCl3, 75.4 MHz): δ = 185.44 (C-14), 183.73 (C-11), 150.7 (C-12), 169.4 (CH_3_CO), 149.9 (C-9), 139.5 (C-8), 124.6 (C-13), 64.5 (C-7), 46.1 (C-5), 41 (C-3), 35.8 (C-1), 32.9 (C-4), 39.1 (C-10), 24.6 (C-6), 32.9 (C-18), 24.1 (C-15), 21.6 (C-19), 21.1 (CH_3_CO), 19.9 (C-17), 19.7 (C-16), 18.5 (C-20), 18.81 (C-2); El-MS: *m/z* (rel int.): 374 [M]^ +^ (5), 332 (100), 314 (95), 298 (74), 267 (56), 227 (58), 183 (24), 83 (14), 43 (34). 

11β-Hydroxymanoyl oxide (**5**): C_20_H_34_O_2_; ^1^H NMR (CDCl_3_, 300.13 MHz): δ = 5.85 (1H, dd, *J* = 17.4, 10.7 Hz, H-14), 5.12 (1H, dd,* J* = 17.4, 1.5 Hz, H-15_trans_), 4.91 (1H, dd, *J* = 10.7, 1.5 Hz, H-15_cis_), 4.38 (1H, ddd,* J* = 9.0, 5.1, 3.2 Hz, H-11), 1.97 (1H, dd,* J* = 14.2, 6.0 Hz, H-12β), 1.84 (1H, dd, *J* = 14.2, 4.7 H-12α), 1.80 (1H, m, H-1β), 1.73 (1H, m, H-7β), 1.66 (1H, m, H-2β), 1.62 (1H, m, H-6β), 1.59 (3H , s, H-17), 1.47 (1H, m, H-7α), 1.44 (1H, m, H-2α), 1.42 (3H, s, H-16), 1.38 (1H, m, H-3β), 1.35 (1H, m, H-6α), 1.31 (1H, d, *J* = 3.9 Hz, H-9), 1.15 (3H, s, H-20), 1.08 (1H , m, H-3α), 0.98 (1H, dd, *J* = 12.5, 3.6 Hz, H-1α), 0.88 (1H, m, H-5), 0.84 (3H, 3, H-18), 0.81 (3H, s, H-19). ^13^C NMR (CDCl_3, _75.4 MHz): δ = 147.7 (C-14), 110.5 (C-15), 74.8 (C-8), 72.4 (C-13), 65.2 (C-11), 57.00 (C-5), 56.4 (C-9), 44.2 (C-12), 44.5 (C-7), 41.9 (C-3), 39.2 (C-1), 37.7 (C-10), 33.5 (C-18), 33.2 (C-4), 29.7 (C-16), 27.4 (C-17), 21.4 (C-19) 20.1 (C-6), 18.4 (C-2), 17.4 (C-20); El-MS: *m/z *(rel int.): 306 [M]^ +^ (2), 291(36), 273 (64), 255(31), 223 (83), 177 (33), 137 (39), 81 (45), 69 (62), 55 (75), 43 (100). 

Microstegiol (**6**): C_20_H_26_O_2_; ^1^H NMR (CDCl_3_, 300.13 MHz): δ = 7.06 (1H, d, *J* = 7.6 Hz, H-6), 6.96 (1H, br. s, H-14), 6.89 (1H, d,* J* = 7.6 Hz, H-7), 4.52 (1H, s, 11-OH), 3.59 (1H, ddd,* J* = 14.3, 12.2, 2.4 Hz, H-1β), 3.01 (1H , heptet, *J* = 6.9 Hz, H-15), 2.78 (1H, ddd,* J* = 14.2, 6.5, 2.4 Hz, H-1α), 2.38 (1H, m, H-3α), 2.38 (3H, s, H-20), 1.79 (1H, m, H-2α), 1.43 (1H , m, H-2β), 1.26 (1H, dt, *J* = 14, 4.0 Hz, H-3β), 1.20 (3H, d, *J* = 6.9 Hz, H-17), 1.15 (3H, d, *J* = 6.9 Hz, H-16), 0.79 (3H, s, H-19), 0.78 (3H, s, H-18). 13C NMR (CDCl3, 75.4MHz): δ = 206.2 (C-12), 143.2 (C-10), 141.0 (C-13), 140.9 (C-14), 139.3 (C-9), 137.3 (C-5), 130.1 (C-6), 129.0 (C-8), 126.7 (C-7), 84.3 (C-11), 42.9 (C-3), 39.0 (C-4), 28.0 (C-19), 27.0 (C-15), 26.8 (C-1), 23.5 (C-2), 22.1 (C-17/C-16), 21.6 (C-18), 21.4 (C-20), 21.1 (C-16/C-17); El-MS: *m/z *(rel int.): 298 [M]^ +^ (73), 229 (100), 201 (17), 165 (7), 141 (8), 128 (7), 115 (6), 91 (2), 69 (2), 41 (4).

I-keto-aethiopinone (**7**): C_20_H_22_O_3_; ^1^H NMR (CDCl_3_, 300.13 MHz): δ = 7.47 (1H, d, *J* = 7.6 Hz, H-7), 7.23 (1H, d, *J* = 7.6 Hz, H-6), 7.15 (1H, s, H-14), 4.73 (1H, q,* J* = 1.6 Hz, H-18a), 4.69 (1H, s, H-18b), 3.05 (1H, heptet, *J* = 6.8 Hz, H-15), 2.61 (4H, m, H-2,3), 2.26 (3H, s, H-20), 1.77 (3H, s, H-19), 1.26 (2H, td,* J* = 6.1, 5.2, 2.3 Hz, H-3), 1.18 (6H, d, *J* = 6.8 Hz, H-16,17). ^13^C NMR (CDCl_3_, 75.4 MHz): δ = 206.4 (C-1), 179.8 (C-12), 179.4 (C-11), 146.1 (C-4), 146.1 (C-13), 145.8 (C-10), 144.6 (C-5), 138.5 (C-14), 137.9 (C-6), 135.7 (C-9), 133.7 (C-8), 129.6 (C-7), 109.9 (C-18), 41.4 (C-2), 31.1 (C-3), 27.0 (C-15), 22.8 (C-19), 21.5 (C-16), 21.52 (C-17), 18.5 (C-20); El-MS: *m/z *(rel int.): 310 [M]^ +^ (2), 291 (2), 255 (12), 216 (50), 165 (100), 119 (42), 91 (20), 67 (33), 41 (47). 

14-deoxycoleon U (**8**): C_20_H_26_O_4_; ^1^H NMR (CDCl_3_, 300.13 MHz): δ = 7.71 (1H, s, H-14), 7.11 (1H, s, 6-OH), 3.10 (1H, heptet, *J* = 6.9 Hz, H-15), 2.97 (1H, ddd,* J* = 13.6, 6.6, 2.3 Hz, H-1β), 2.02 (1H, m, H-3α), 1.86 (1H, ddd,* J* = 13.5, 6.6, 3.9 Hz, H-2β), 1.75 (1H, H-2α), 1.67 (3H, s, H-20), 1.57 (1H, m, H-1α), 1.46 (3H, s, H-19), 1.45 (3H, s, H-18), 1.38 (1H, m, H-3β), 1.29 (3H, d, *J *= 6.9 Hz, H-16), 1.16 (3H, d, *J* = 6.9 Hz, H-17). ^13^C NMR (CDCl_3_, 75.4 MHz): δ = 179.9 (C-7), 145.4 (C-12), 143.4 (C-5), 142.9 (C-11), 141.0 (C-6), 138.2 (C-9), 132.8 (C-13), 120.9 (C-8), 116.4 (C-14), 40.7 (C-10), 36. (C-4), 36.4 (C-3), 30.2 (C-1), 27.9 (C-18), 27.8 (C-20), 27.3 (C-15), 27.1 (C-19), 22.6 (C-16), 22.4 (C-17), 17.8 (C-2); El-MS: *m/z *(rel int.): 330 [M] ^+^ (57), 315 (9), 287 (14), 260 (100), 245 (22), 217 (19), 191 (6), 115 (5), 91(4), 43 (5). 

*Molecular docking study*

Docking studies were carried out by AutoDock Tools 1.5.4 program (ADT) molecular simulation software. The X-ray crystallographic structure of the human DNA topoisomerase I (topo I) was downloaded from RCSB protein data bank (PDB code: 1k4t) for docking study. Co-crystallized ligand and water molecules were removed from crystal structures. Then, all polar hydrogen atoms were added and Kollman charges were assigned to the proteins and saved in pdbqt format by ADT. The grid map was determined based on the coordinates of native ligand (topotecan) in X-ray crystal structure. The energy minimized compounds were docked to active site of topo I using the Lamarkian genetic algorithm with grid sizes 60 × 60 × 60 (grid spacing 0.375 Å), the maximum number of evaluations were set to 2.5 × 10^6^, the number of GA runs were 100, and the maximum number of generations were set as 27,000, and all of the other options were set as default. For interpretation of docking result, the conformation with lowest binding free energies from the largest population cluster was selected. 

*Validation of docking*

Performance and validation of docking study was evaluated by self-docking of the native ligand into the 1k4t active site. The self-docking of native ligand into active pockets of receptor showed a binding free energies of −11.64 kcal/mol with RMSD of 0.846 Å. According to acceptable RMSD of self-docking (< 2 Å), the obtained result indicates that the docking protocol was valid for topo I docking system.

*Ligands preparation*

The 3D structures of purified compounds were taken from PubChem data bank in SDF format (http://pubchem.ncbi.nlm.nih.gov). Gaussian 09 program was applied to minimize the ligands for docking purpose. Geometries of compounds were optimized using density functional theory (DFT) at B3LYP level of theory with the 6-31G (d) as general basis set in gas phase and the outputs of Gaussian were saved in pdb format. The vibrational frequency analysis was performed at the same level to check that there are no imaginary frequencies in minimized structures. Then, the gasteiger charges were added by ADT and saved in pdbqt format for docking study.

*Calculation of molecular physicochemical properties*

To evaluate purified compounds as drug candidate, some molecular properties such as octanol-water partition coefficient (log P), number of H-bond donors (HBD), number of H-bond acceptors (HBA), and molar refractivity (MR) were calculated using a freely accessible web-server (http://www.scfbio-iitd.res.in/software/drugdesign/lipinski.jsp) and also number of rotatable bonds (RB), and topological polar surface area (tPSA) were obtained from Pub Chem data bank for each compounds (Table 1).

## Results and Discussion

**Figure 1:**
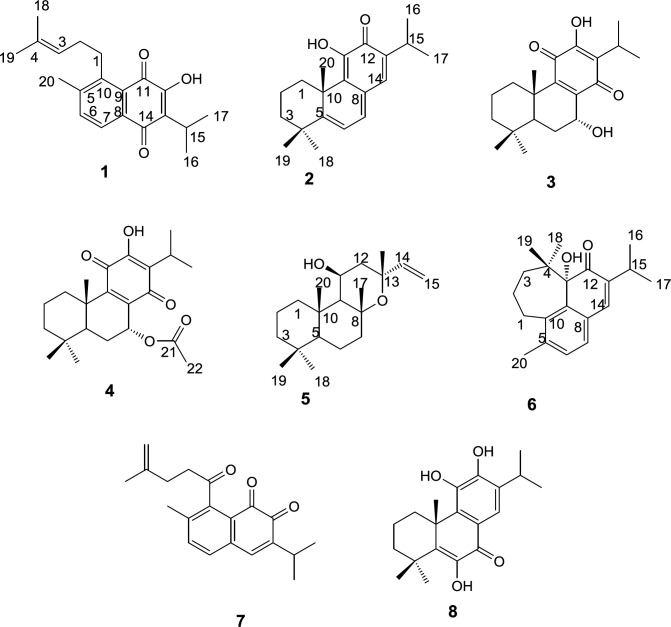
Structures of purified compounds

Preparative RP-HPLC of more polar fractions obtained from normal-phase open column chromatography of the roots DCM extracts of *S. lachnocalyx* led to characterization of eight further known diterpenoids (Figure 1); 12-hydroxysapriparaquinone (**1**), 15-deoxyfuerstione (**2**), horminon (**3**), 7α-acetoxyroyleanone (**4**), 11β-hydroxymanoyl oxide (**5**), microstegiol (**6**), 1-keto-aethiopinone (**7**) and 14-deoxycoleon U (**8**).

 The structure of the compounds were elucidated using the spectroscopic data including; EI-MS, ^1^H and ^13^C NMR and by comparing them with those reported in the literature for authentic compounds. The ^1^H NMR, and ^13^CNMR spectroscopic data obtained for compounds **1 **and** 7 **were compatible with the rearranged abietane diterpenoid skeleton, showing characteristic peaks for H(C)-6 (δ_H_ 7.51 and 7.23), H(C)-7 (δ_H_ 7.96 and 7.47), H(C)-15 (δ_H_ 3.36 and 3.05), 16, 17 and H(C)-18 (δ_H_ 1.30, 1.30, 1.73 and 1.18, 1.18, 4.73, 4.69), 19 (δ_H_ 1.61 and 1.77) which were in good agreement with those reported in the literature for 12-hydroxysapriparaquinone and 1-keto-aethiopinone, respectively (11, 12). On the other hand, the NMR spectroscopic data of compounds **2, 3, 4** and **8 **were quite similar to those reported for 15- deoxyfuerstione, horminon, 7α-acetoxyroyleanone, and 14-deoxycoleon U with an abietane diterpene characteristic signals. The quaternary methyl H(C)-20 signal appeared at δ_H_ 1.56, 1.20, 1.23 and 1.67 ppm, for compounds **2, 3, 4**, and **8** respectively. The rest recorded signals, especially the methyls signals of H(C)-16-19 were in good agreements with those compounds that were isolated from *Salvia moorcraftian *(*S. moorcraftian*)*, Salvia sahendica *(*S. sahendica*)*, Salvia rhytidea *(*S. rhytidea*) and* Salvia broussonetii *(*S. broussonetii*) respectively (13-16). In the process of structure elucidation of compound **5**, the characteristic peaks of H(C)-14, 15 olefinic signal at δ_H_ 5.85, 5.12, 4.91 and two methyls of H(C)-16, 17 at δ_H_ 1.42, 1.59 ppm, respectively, together with the EIMS data guided us to the labdane-type diterpenoid which has been isolated from *Salvia candidissima *(*S. candidissima*)*,* previously (17). Based on MS,^ 1^H and ^13^C-NMR data, compound **6 **was identified as a rearranged abietane diterpenoid that unlike the seco-4,5-abiatane a new cycloheptane ring was formed with a C-4/ C-11 bond formation, the resulting compound showed two germinal methayl signals at δ_H_ 0.78, 0.79 H(C)-18, 19 were in agreement with the NMR data reported for microstegiol isolated from *Salvia microstegia* (*S. microstegia),* but upfield shifted in comparison to the respective signals of the abiatane diterpenoids (18). 

Compounds **2**, **3**, **5**, **6,** and **8** were tested against human breast adenocarcinoma (MCF-7) and human chronic myelogenous leukemia (K562) cell lines, while the remaining compounds were not tested because of the lack of quantity (Table 2). Several natural diterpenoids, especially abietane and labdan types have been investigated for their cytotoxic potential and have shown different levels of cytotoxicity against various cell lines. For instance, the cytotoxic activity of compounds **2**, **5,** and **8 **against two selected cell lines and compounds **3** and **6 **against K562 are being evaluated for the first time in the current study. Compound **2**, **3**, **6,** and **8** exhibited cytotoxic effect with IC_50_ values in the range of 2.63-11.83 µg/mL, which seemed as considerable activities as compared to cisplatin, a standard chemotherapeutic agent with IC_50 _values of 12.49 and 2.91 µg/mL against MCF-7 and K562 cells, respectively. Compounds **2**, **3**, **6**, and **8** showed higher cytotoxic activities compared to **5. **The higher activity of the abietane diterpenoids (about 10 times greater) can be related to the presence of *α,β*-unsaturated carbonyl moiety in their structures or the higher cytotoxicity of abietane compared to labdane diterpenoids (19).

One of the most important drug targets in cancer therapy is topo I, which plays a unique role in DNA replication and cell division. Inhibition of topo I function results in DNA damage that ultimately leads to cell death. Therefore, topo I is considered as a therapeutic target for cancer chemotherapy (20). In a previous report, 324 natural compounds were screened for their capacity in topo I inhibition and among them 7-ketoroyleanone with abietane diterpenoid skeleton exhibited topo I inhibitory activities (21). Fronza and coworkers evaluated the inhibitory effects of some abietane diterpenoids such as 7α-acetoxyroyleanone, horminone, royleanone, 7-ketoroyleanone and sugiol on human DNA topo I. They showed that the abietane diterpenoids acted as topo I inhibitors in comparison to camptothecin, a positive control (22). According to the above mentioned report, topo I inhibition can be considered as possible mechanism for cytotoxicity of the purified compounds with abietane diterpenoids skeleton. Therefore, the above phenomenon is checked by molecular docking calculations.

**Figure 2:**
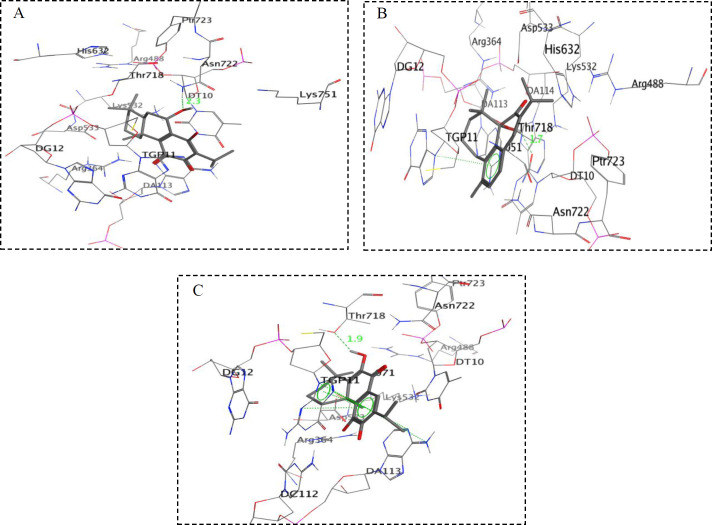
Simplified structures of docking results of (A) compound **3**, (B) compound **6 **and (C) compound **8 **with topoisomerase I active site (PDB: 1k4t)

In order to assess the interactions of antiproliferative compounds with topo I; compounds **2**, **3**, **5**, **6,** and **8 **were docked into the active pocket of topo I. Docking results of the purified compounds are presented in Table 1 as free energy of binding (ΔG_b_) and K_i _(inhibition constant). Among the tested compounds, the compound** 3** exhibited the best docking score with free binding energy of -8.30 kcal/mol and formed a hydrogen bond with a distance of 2.3 Å apart from the Asn722 amino acid residue of the topo I active site (Figure 2A). On the other hand, compound **6** demonstrated free binding energy of -8.22 kcal/mol and formed a hydrogen bond with DT10 in distance of 1.7 Å besides a stacking interaction with TGP11 (Figure 2B). Compound **8** with free binding energy of -8.03 kcal/mol showed an H-bond with Thr718 at a distance of 1.9 Å besides two stacking with DA113 and TGP11 (Figure 2C). 11-OH and 12 C=O groups in compound **2** formed two H-bonds with TGP11 at distances of 2.2 and 1.9 Å, respectively and caused a free binding energy of -8.01 kcal/mol. 

Different papers have demonstrated various orientations in active site of topo1 in interaction with their inhibitors (23-26). These differentiations could be described based on molecular properties of topo I inhibitors. However, in all of them the H-bonding and stacking interaction between topo1 inhibitors with the DNA bases and critical residues of active site such as DA113, TGP11, Asn722, Lys532, Asp533, Arg364, Asn352, Tyr723 have been reported as the key interactions. Based on the obtained data, it was found that compounds **2**, **3**, **6,** and **8 **showed a good fitting in the active site of topo I, having docking scores between -8.01 to -8.30 kcal/mol compared to compound **5** with the score of -6.93 kcal/mol.

**Table 1:** Results of virtual modeling study and molecular properties calculated for cytotoxic compound

**Compounds**	**ΔG**_b_ ** (kcal/mol) (PDB** **: 1k4t)**	**Ki (μM)**	**Log p**	**Molecular mass**	**HBD**	**HBA**	**MR**	**tPSA (Å** ^2^ **)**	**RB**
**2**	-8.01	0.975	5.04	298	1	2	89.48	37.3	1
**3**	-8.30	0.633	3.5	332	2	4	90.93	74.6	1
**5**	-6.39	7.310	4.71	306	1	2	90.66	29.5	1
**6**	-8.22	0.735	4.16	298	1	2	89.61	37.3	1
**8**	-8.03	0.695	4.69	330	3	4	92.82	77.8	1

**Table 2:** Anti-proliferative effects of diterpenoids isolated from roots of *S. lachnocalyx* against MCF-7 and K-562 cell lines

**IC**_50_ ** (μg/mL)**						
**Compound**	**2**	**3**	**5**	**6**	**8**	**Cisplatin**
K562	4.70 ± 0.20	9.6 ± 0.77	27.48 ± 0.97	3.30 ± 0.21	2.63 ± 0.10	2.91 ± 0.08
MCF-7	5.13 ± 0.24	11.8 3 ± 0.24	24.75 ± 1.07	4.67 ± 0.35	2.70 ± 0.09	12.49 ± 1.25

Values are presented as mean ± SEM. of 3–5 experiments. Cisplatin was tested as a reference cytotoxic agent.

## Conclusion

The above mentioned docking results were in agreement with the order of higher cytotoxic activity of the isolated compounds, **2, 3, 6,** and **8** with an abietane or rearranged carbon skeletons, while compound** 5** with a labdane type skeleton has less cytotoxic activity. Therefore, based on docking results, the inhibition of topo I can be considered as a mechanism for the cytotoxicity of the tested active compounds.

The isolated active compounds possessed drug-like features as expressed in Lipinski’s rule-of-five including log P ≤ 5, MW ≤ 500, hydrogen bond donor (HBD) ≤ 5, hydrogen bond acceptor (HBA) ≤ 10, and molar refractivity (MR) between 40-130 (29, 30). Furthermore, the compounds had rotatable bonds ≤ 10 and polar surface area ≤ 140 Å^2^. Therefore, the abietane diterpenoids with remarkable cytotoxic potential could be considered as drug candidates for more advanced drug discovery phases. 

## References

[1] Jassbi AR, Zare S, Firuzi O, Xiao J (2016). Bioactive phytochemicals from shoots and roots of Salvia species. Phytochem. Rev..

[2] Asadollahi M, Firuzi O, Heidary Jamebozorgi F, Alizadeh M, Jassbi AR (2019). Ethnopharmacological studies, chemical composition, antibacterial and cytotoxic activities of essential oils of eleven Salvia in Iran. J. Herbal Med..

[3] Hadavand Mirzaei H, Firuzi O, Baldwin IT, Jassbi AR (2017). Cytotoxic activities of different iranian solanaceae and lamiaceae plants and bioassay-guided study of an active extract from Salvia lachnocalyx. Nat. Prod. Commun..

[4] Jamzad Z, Assadi M, Maassoumi, AA,, Mozaffarian V (2012). Lamiaceae. Flora of Iran.

[5] Farimani MM, Mazarei Z (2014). Sesterterpenoids and other constituents from Salvia lachnocalyx Hedge. Fitoterapia.

[6] Mirza M, Bahernik Z (2007). Extraction and identification of the essential oil components of Salvia lachnocalyx Hedge. Iran. J. Med. Aromat. Plants.

[7] Mirzaei HH, Firuzi O, Schneider B, Baldwin IT, Jassbi AR (2017). Cytotoxic diterpenoids from the roots of Salvia lachnocalyx. Rev. Bras. Farmacogn..

[8] Chen X, Ung CY, Chen Y (2003). Can an in-silico drug-target search method be used to probe potential mechanisms of medicinal plant ingredients? Nat. Prod. Rep..

[9] Osguthorpe DJ, Sherman W, Hagler AT (2012). Generation of receptor structural ensembles for virtual screening using binding site shape analysis and clustering. Chem. Biol. Drug Des..

[10] Firuzi O, Miri R, Asadollahi M, Eslami S, Jassbi AR (2013). Cytotoxic, antioxidant and antimicrobial activities and phenolic contents of eleven Salvia species from Iran. Iran. J. Pharm. Res..

[11] Topcu G, Eriş C, Ulubelen A (1996). Rearranged abietane diterpenes from Salvia limbata. Phytochemistry.

[12] Michavila A, María C, Rodríguez B (1986). 20-Nor-abietane and rearranged abietane diterpenoids from the root of Salvia argentea. Phytochemistry.

[13] Simões F, Michavila A, Rodríguez B, Maria C, Hasan M (1986). A quinone methide diterpenoid from the root of Salvia moorciuftiana. Phytochemistry.

[14] Jassbi AR, Mehrdad M, Eghtesadi F, Ebrahimi SN, Baldwin IT (2006). Novel rearranged abietane diterpenoids from the roots of Salvia sahendica. Chem. Biodivers..

[15] Jassbi AR, Eghtesadi F, Hazeri N, Ma’sumi H, Valizadeh J, Chandran JN, Schneider B, Baldwin IT (2017). The roots of Salvia rhytidea: a rich source of biologically active diterpenoids. Nat. Prod. Res..

[16] Fraga BM, Díaz CE, Guadaño A, González-Coloma A (2005). Diterpenes from Salvia broussonetii transformed roots and their insecticidal activity. J. Agric. Food Chem..

[17] Topcu G, Tan N, Ulubelen A, Sun D, Watson W (1995). Terpenoids and flavonoids from the aerial parts of Salvia candidissima. Phytochemistry.

[18] Ulubelen A, Topcu G, Tan N, Lin LJ, Cordell GA (1992). Microstegiol, a rearranged diterpene from Salvia microstegia. Phytochemistry.

[19] Amslinger S (2010). The Tunable functionality of α, β-unsaturated carbonyl compounds enables their differential application in biological systems. Chem. Med. Chem..

[20] Liu LF (1989). DNA topoisomerase poisons as antitumor drugs. Annu. Rev. Biochem..

[21] Han HJ, Tan NH, Zeng GZ, Fan JT, Huang HQ, Ji CJ, Jia RR, Zhao QS, Zhang YJ, Hao XJ, Wang LQ (2008). Natural inhibitors of DNA topoisomerase I with cytotoxicities. Chem. Biodivers..

[22] Fronza M, Lamy E, Günther S, Heinzmann B, Laufer S, Merfort I (2012). Abietane diterpenes induce cytotoxic effects in human pancreatic cancer cell line MIA PaCa-2 through different modes of action. Phytochemistry.

[23] Ioanoviciu A, Antony S, Pommier Y, Staker BL, Stewart L, Cushman M (2005). Synthesis and mechanism of action studies of a series of norindenoisoquinoline topoisomerase I poisons reveal an inhibitor with a flipped orientation in the ternary DNA-enzyme-inhibitor complex as determined by X-ray crystallographic analysis. J. Med. Chem..

[24] Staker BL, Feese MD, Cushman M, Pommier Y, Zembower D, Stewart L, Burgin AB (2005). Structures of three classes of anticancer agents bound to the human topoisomerase I-DNA covalent complex. J. Med. Chem..

[25] Staker BL, Hjerrild K, Feese MD, Behnke CA, Burgin AB, Stewart L (2002). The mechanism of topoisomerase I poisoning by a camptothecin analog. Proc. Natl. Acad. Sci..

[26] Lauria A, Ippolito M, Almerico AM (2007). Molecular docking approach on the Topoisomerase I inhibitors series included in the NCI anti-cancer agents mechanism database. J. Mol. Model..

